# A Whole-Process Visible Strategy for the Preparation of *Rhizomucor miehei* Lipase with *Escherichia coli* Secretion Expression System and the Immobilization

**DOI:** 10.1186/s12934-024-02432-y

**Published:** 2024-05-27

**Authors:** Mingjun Yang, Xianhui Su, Jun Yang, Zhiwen Lu, Jie Zhou, Fei Wang, Yang Liu, Lixin Ma, Chao Zhai

**Affiliations:** State Key Laboratory of Biocatalysis and Enzyme Engineering, School of Life Sciences, Hubei University, Wuhan, 430062 People’s Republic of China

**Keywords:** RM lipase, sfGFP, Secretion expression, Immobilization

## Abstract

**Background:**

*Rhizomucor miehei* (RM) lipase is a regioselective lipase widely used in food, pharmaceutical and biofuel industries. However, the high cost and low purity of the commercial RM lipase limit its industrial applications. Therefore, it is necessary to develop cost-effective strategies for large-scale preparation of this lipase. The present study explored the high-level expression of RM lipase using superfolder green fluorescent protein (sfGFP)-mediated *Escherichia coli* secretion system.

**Results:**

The sfGFP_(−15)_ mutant was fused to the C-terminus of RM lipase to mediate its secretion expression. The yield of the fusion protein reached approximately 5.1 g/L with high-density fermentation in 5-L fermentors. Unlike conventional secretion expression methods, only a small portion of the target protein was secreted into the cell culture while majority of the fusion protein was still remained in the cytoplasm. However, in contrast to intracellular expression, the target protein in the cytoplasm could be transported efficiently to the supernatant through a simple washing step with equal volume of phosphate saline (PBS), without causing cell disruption. Hence, the approach facilitated the downstream purification step of the recombinant RM lipase. Moreover, contamination or decline of the engineered strain and degradation or deactivation of the target enzyme can be detected efficiently because they exhibited bright green fluorescence. Next, the target protein was immobilized with anion-exchange and macropore resins. Diethylaminoethyl sepharose (DEAE), a weak-basic anion-exchange resin, exhibited the highest bind capacity but inhibited the activity of RM lipase dramatically. On the contrary, RM lipase fixed with macropore resin D101 demonstrated the highest specific activity. Although immobilization with D101 didn’t improve the activity of the enzyme, the thermostability of the immobilized enzyme elevated significantly. The immobilized RM lipase retained approximately 90% of its activity after 3-h incubation at 80 °C. Therefore, D101 was chosen as the supporting material of the target protein.

**Conclusion:**

The present study established a highly efficient strategy for large-scale preparation of RM lipase. This innovative technique not only provides high-purity RM lipase at a low cost but also has great potential as a platform for the preparation of lipases in the future.

**Supplementary Information:**

The online version contains supplementary material available at 10.1186/s12934-024-02432-y.

## Background

Lipases (glycerol ester hydrolases E.C. 3.1.1.3) catalyze hydrolysis as well as esterification reactions and is one of the most widely used enzymes in biocatalysis being identified [1, 2]. In terms of regioselectivity, there are three types of lipases: *sn*-1,3-selective (hydrolyze the ester bonds of triglycerides at positions R1 or R3), *sn*-2-selective (hydrolyze the ester bonds of triglycerides at position R2) and non-selective. Lipases derived from *R. miehei* (RM lipase) is a regioselective lipase which can only catalyze 1, 3-diglceride or 1-monoglyceride. It can catalyze the hydrolysis of triacylglycerols (TAGs) into glycerol and fatty acids, synthesize esters from various alcohols and fatty acids and undergo transesterification and aminolysis reactions [3]. Therefore, it is widely used in chemical synthesis [4, 5], degradation of biological waste oil [6], food [7–9] and biodiesel [6, 10]. RM lipase is commercially available in both soluble (e.g., Palatase 2000 L of Novozyme, Danmark)and immobilized forms (e.g., Lipozyme RM IM of Novozyme, Danmark [3, 11]. However, the high price of the commercial lipases limits their industrial-scale applications. Therefore, it is urgent to develop strategies for large-scale preparation of RM lipase at low cost.

RM lipase is a single-chain α/β type protein of 269 amino acid residues. Novozyme expressed RM lipase with genetic modified *Aspergillus oryzae*. RM lipase was also expressed with *Pichia pastoris* [12, 13] or through yeast cell surface display of *Pichia pastoris* [14]. However, the yield was relatively low. For instance, only 0.77 mg/mL RM lipase was obtained with *P. pastoris* secretion expression using engineered strain bearing two copies of the target gene [12]. Recombinant lipases can also be expressed with *E. coli* [15]. However, the intracellular expression of the target protein requires multi-step of purification before further used in food and pharmaceutical industries. Therefore, many strategies have been developed to achieve secretion expression of heterologous proteins in *E. coli* [16]. We reported a novel *E. coli* secretion expression system mediated by sfGFP previously [17]. It indicated that sfGFP motif guided the secretion of various proteins fused at its C-terminus. It proposed that the β-barrel structure and negative charges distributed on the surface of the molecules facilitated the secretion of the target protein fused with it. Therefore, serial of sfGFP bearing various net negative charges were generated. Among them, sfGFP mutant bearing 15 net negative charges, sfGFP_(-15)_ demonstrated the highest efficiency. In the present study, we discovered that RM lipase can be expressed with this *E. coli* secretion system efficiently. Interestingly, RM lipase was secreted efficiently with the target protein fused to either N- or C-terminus of sfGFP_(-15)_, which suggested that sfGFP secretion system works with a mechanism different from the conventional signal peptides. The recombinant protein was further immobilized with resins and used to convert TAG to diacylglycerol (DAG).

**Fig. 1:**
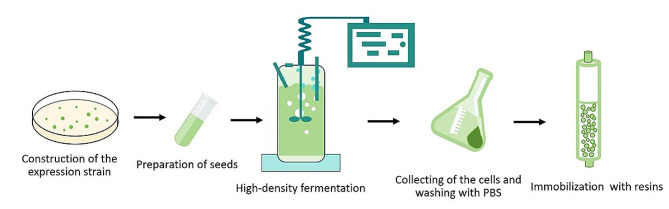
Schematic illustrating the large-scale preparation of the immobilized RM lipase using *E. coli* secretion expression system mediated by sfGFP_(−15)_.

The secretion expression of RM lipase levitated the cellular toxicity of the protein and simplified the downstream purification, hence, increased the yield of the target protein and reduced the overall cost of the protein preparation. More importantly, the whole procedure can be monitored by naked eyes because sfGFP_(-15)_ endows the engineering strains and the enzyme obvious green colour (Fig. 1). Therefore, the contamination or decline of the engineering strain can be detected by naked eyes easily. The fermentation progress can be monitored through measuring the fluorescence intensity of the cell culture. The activity of the fixed enzyme can be estimated by the colour change of the resins.

## Materials and Methods

### Strains, Plasmids, Media and Reagents

*E. coli* DH5α for gene cloning were stored in our lab. *E. coli* BL21, BL21 (DE3), BL21 (DE3) pLysS, BL21 CodonPlus (DE3) were purchased from TransGen Biotechnology (China). *E. coli* C41, C43 and Rossetta (DE3) were purchased from Biyotime (China). Plasmid pET23a-sfGFP_(−15)_ was stored in our lab. Luria–Bertani (LB) were prepared as described in the Manual of Molecular Cloning [18]. The substrate 4-nitrophenyl palmitate (p-NPP) was purchased from Macklin (USA) and 4-Nitrophenol was purchased from Macklin (USA). Dibutyl phthalate (DBP), bis(2-ethylhexyl) phthalate (DEHP), dicyclohexyl phthalate (DCHP) were purchased from ANPEL-Trace Standard Technical Services (Shanghai, China). Isopropyl cyclohexane-3-ene carboxylate (CHCI) and methyl cyclohex-3-ene-1-carboxylate (CHCM) were purchased from Haohong Scientific Co. Ltd (Shanghai, China). XAD-22 resin was purchased from ROHM (USA). D101, D201, AB-8 and D301 resins were purchased from Anhui Sanxing (China). DEAE resin was purchased from Ruidahenghui (China). DAG standard was purchased from Yuanye Biotechnology (China). All other chemicals were analytical reagents.

### Expression and Recovering of RM Lipase Fused with sfGFP_(−15)_

The ORF encoding RM lipase was synthesized by Sangon (China) and cloned into pET23a vector through TLTC method to generate expression vectors pET23a-RM, pET23a-sfGFP_(−15)_-RM and pET-RM-sfGFP_(−15)_ [19]. To facilitate the purification, a 6×His tag was fused to the N-termini of the fusion proteins. The recombinant plasmids were verified by Sanger sequencing, followed by transforming into *E. coli* BL21 (DE3). To induce the expression of the target genes, the transformants were incubated at 37 °C to OD_600_ of 0.6–0.8, and then induced with 0.5 mM of Isopropyl-β-D-thiogalactopyranoside (IPTG) at 18 °C for 24 h. To recover the recombinant protein, the cells were collected by centrifugation at 5000 rpm for 10 min, followed by incubating with 1 mL of PBS at 4 °C, overnight.

### Purification of RM-sfGFP_(−15)_ with Ni-affinity Chromatography

Cells were collected and resuspended in lysis buffer (50 mM Tris-HCl; 200 mM NaCl; 50 mM NaH_2_PO_4_; 25 mM Imidazole; 10% Glycerol, pH 8.0) with lysozyme to a final concentration of 1 mg/mL. Samples were ultrasonicated to break the cells. The crude cell lysate was collected after centrifugation at 13,000 rpm for 10 min. The sample was then centrifugated at 13,000 rpm for 10 min, and the supernatant was applied to Ni-NTA beads for affinity purification. The column was washed twice with 2 column volume of wash buffer (50 mM Tris-HCl; 200 mM NaCl; 50 mM NaH_2_PO_4_; 40 mM Imidazole, pH 8.0). One column volume of elution buffer (50 mM Tris-HCl; 200 mM NaCl; 50 mM NaH_2_PO_4_; 200 mM Imidazole, pH 8.0) was used to recover the target protein. The sample was then collected and dialyzed with a Millipore 10-kDa cut-off membrane at 4 °C to remove ions and salts, followed by resuspending with storage buffer (50 mM Tris-HCl, pH 7.5).

### Evaluation of the Concentration of the Target Protein

To determining the concentration of RM lipase fused with sfGFP_(−15)_ in the cells directly, the fusion protein was purified and the concentration was evaluated with Bradford method [20]. Meanwhile, the fluorescence intensity of serial diluted fusion protein was measured with spectrum photometer at wavelength of 488 nm and a standard curve was plot.

### Sodium Dodecyl Sulfate Polyacrylamide Gel Electrophoresis (SDS-PAGE)

Protein samples were separated via SDS-PAGE using 10 ~ 15% (w/v) polyacrylamide gel, followed by staining with Coomassie Brilliant Blue G250.

### Analysis of the Enzymatic Activity of RM Lipase

To investigate the activity of the recombinant RM lipase, p-NPP was used as the substrate. In a standard assay, the reaction mixture contained 40 µL of p-NPP (12.5 mM), 940 µL of Tris-HCl buffer (50 mM, pH 8.0) and 20 µL of diluted enzyme solution. The mixture was incubated at 40 ℃ for 5 min and the reaction was terminated with 60 µL of 20% Trichloroacetic Acid. Subsequently, 300 µL of 0.5 M NaOH was added and the sample was centrifuged at 1000 g for 1 min, followed by measuring the absorbances at 405 nm. All experiments were conducted in triplicate and 4-Nitrophenol was used to plot the standard curve. One unit (U) of lipase activity was defined as the amount of enzyme that released 1 µmol of p-NP from p-NPP per min under the assay conditions.

To analyze the substrate spectrum of RM lipase with or without sfGFP_(−15)_, several commonly used esters, including CHCM, CHCI, DBP, DEHP, and DCHP were tested. To set the reactions, 50 µL of CHCM or CHCI (1 M), 28 µL of DBP (70mM, dissolved in acetonitrile), 40 µL DEHP (50 mM), 20 µL of DCHP (100 mM) was used. All the substrates were dissolved in acetonitrile. Equal molar of RM lipase with or without sfGFP_(−15)_ (approximately 7 nmol) was added the reaction mixture to a final volume of 1 mL. The mixture was incubated at 37 ℃ for 40 min, followed by adding 200 µL of acetonitrile to terminate the reaction. The samples were centrifuged at 10,000×g for 1 min, followed by HPLC to analyze the decrease of the substrates.

A Prominence Ultra-Fast Liquid Chromatograph (Shimadzu) equipped with an GL Sciences InertSustain C18 column (4.6 × 250 mm, 5 μm). To analyze CHCM and CHCI, the mobile phase was 10 mM Na_2_HPO_4_ (pH 3.0) and acetonitrile at a flow rate of 1.0 mL/min. The elution condition was 0–15 min with 20–75% (v/v) methanol linear gradient and 15–25 min with 75 − 20% methanol linear gradient. The effluent was monitored at a wavelength of 205 nm. To analyze DBP, DEHP, and DCHP, the mobile phase was 40% acetonitrile and methanol/acetonitrile solution (6:4 in volume) at a flow rate of 1.3 mL/min. The elution program is set to increase the volume fraction of mobile phase B from 0 to 40% (v/v) within the first 0–21 min, from 40 to 60% (v/v) within 21–45 min, from 60 to 80% (v/v) within 45–50 min, from 80 to 100% (v/v) within 50–67 min, and from 100 to 0% (v/v) within 67–68 min. The effluent was monitored at a wavelength of 242 nm. All experiments were conducted in triplicate and the substrates were used to plot the standard curve.

### High-density Fermentation of the Recombinant RM Lipase

Fed-batch fermentation was performed as previously described [21]. The recombinant *E. coli* strain was inoculated into individual flasks containing 100 mL of LB supplemented with 100 µg/mL of Ampicillin, and the cultures were incubated at 200 rpm, 37 °C until OD_600_ reached 0.6. The cell culture was then transferred into 5-L fermenters (BG-4, Baoxing, China) containing 2 L of LB medium (Tryptone 10%, yeast extract 5%, NaCl 10%, pH7.0). During the early stage of the fermentation, the culture was maintained at 37 °C, pH 6.5-7.0 using NaOH. The cell culture was agitated at 300 rpm to maintain 30% dissolved oxygen (DO). After approximately 18 to 24 h, medium (280.5 g/L glucose, 96.2 g/L NH_4_Cl)was added to the cell growth continuously, with DO kept above 20%. When OD_600_ increased to approximately 30–40, the temperature of the cell culture was adjusted to 28 °C and the expression of the target gene was induced with IPTG. DO of the cell culture was kept at 20 to 30% with agitation of 600 rpm. This condition was maintained until the end of the fermentation.

### Immobilization of the Recombinant RM Lipase

The resins were rinsed with 95% of ethanol for 24 h and washed with dH_2_O twice, followed by rinsing with 5% (v/v) HCl for 2 h and washed with dH_2_O until pH back to 7.0. Five mil-liter of the enzyme (5 mg/mL) was added to 0.2 g resin. The mixture was incubated at 4 ℃ overnight to fix the enzyme. The immobilized enzyme was collected by centrifuging at 5000×g for 1 min, followed by washing with the washing buffer twice. Subsequently, the immobilized enzyme was lyophilized for 8 h to remove water and stored at 4℃ for further use. To improve the activity of the fixed enzyme with KCl, equal volume of the enzyme was added to 2.5 mL 25% (w/v) of KCl (supplemented with 0.2% (w/v) of sorbitol and 0.2% (w/v) of sucrose) to a final concentration of 2.5 mg/mL, followed by adding 0.2 g resin.

### Recovering and Reusage of the Immobilized RM Lipase

Approximately 1.5 mg of the immobilized RM lipase was added to the reaction mixture contained 400 µL of p-NPP (12.5 mM), 9.6 mL of Tris-HCl buffer (50 mM, pH 8.0). The mixture was incubated at 40 ℃ for 5 min. Subsequently, the immobilized enzyme was recovered though filtration with a vacuum filtration device using filter paper and applied for the next round of reaction.

### Characterization of the Immobilized RM Lipase

To determine the optimum temperature of the immobilized RM lipase, the enzymatic activity was measured at the gradient temperatures with an interval of 5 °C in phosphate buffer (50 mM, pH8.0). The optimum pH was determined by measuring the enzymatic activities in the following buffer: pH 6.0–8.0 (phosphate buffer, 50 mM) and pH 9.0–10.0 (Tris-HCl buffer, 50 mM). To evaluate the thermostability of the immobilized RM lipase, the enzyme was incubated in phosphate buffer (50 mM, pH 8.0) at 70 ℃, 75 ℃ and 80 ℃ for 0–3 h, followed by measuring the residual activity at 40℃. All the assays were performed in triplicate.

### Glycerolysis of TAG in the Solvent-free System Using RM-sfGFP_(−15)_ IM

The enzymatic glycerolysis reaction was carried out as previously reported [22]. The reaction mixture consisted of glycerol and soybean oil at a molar ratio of 5:1, followed by adding 0.5 g of immobilized lipase. The sample was incubated at 50 °C for 3 h. The reaction was stirred using a magnetic stirrer at 180 rpm. Thin-layer chromatography (TLC) with Silica TLC gel 60F10-20 cm from Haiyang Chemical Co., Ltd. (Qingdao, China) was performed to monitor the progress of the reaction. The solvent system contained Hexane, Anhydrous ether, Formic acid at a ratio of 80:20:2 (v/v/v). Iodine was used to stain the gel.

### Fluorescence-activated Cell Sorting (FACS) Assay to Evaluate the Intensity of Green Fluorescence of RM Lipase-sfGFP_(−15)_

To monitor the expression of the GFP fusion protein during the high-density fermentation with FACS, 5 mL of cell culture was collected every 3 h. Cells from each sample were sorted with Flow Cytometer (Beckman Coulter, USA). The intensity of the green florescence was measured with the FITC channel, 525/460 nm band pass.

To test whether the cells maintained intact after the GFP fusion protein was washing off with PBS buffer, the cells were sorted with Flow Cytometer (Beckman Coulter, USA) before and after washing. The intensity of the green florescence was measured with the FITC channel, 525/460 nm band pass.

## Results

### Construction of the Recombinant Strains for the sfGFP_(−15)_-mediated Secretion Expression of RM Lipase

**Fig. 2:**
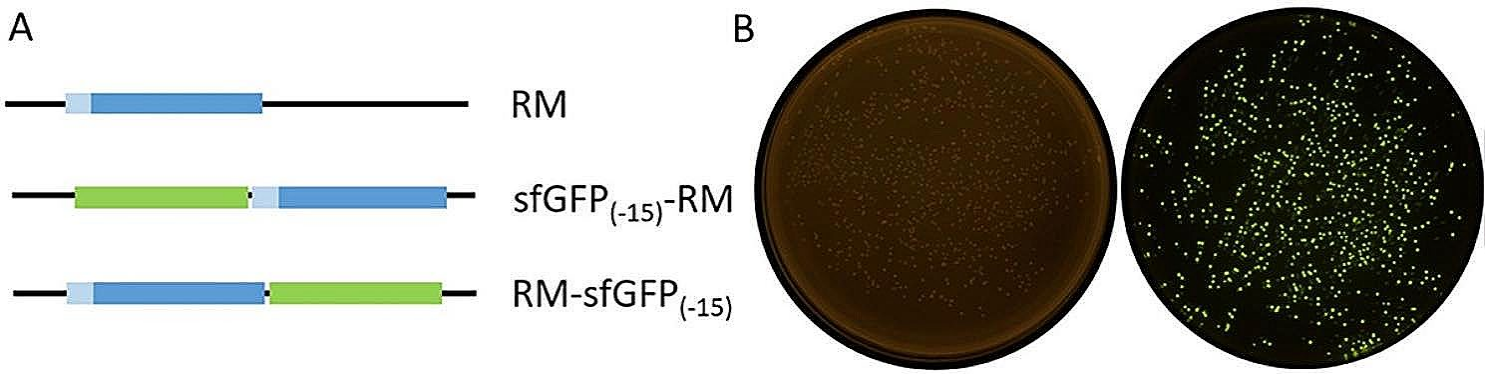
Schematic illustrating the structure of the proteins. (A) the structure of the fusion protein. The ORF of the mature lipase is blue and the propeptide is light blue. The ORF of sfGFP_(−15)_ is green. (B) the colonies of *E. coli* BL21(DE3) bearing pET23a-RM (left) and pET23a-RM-sfGFP_(−15)_ (right) on LB plates supplemented with Ampicillin

**Fig. 3:**
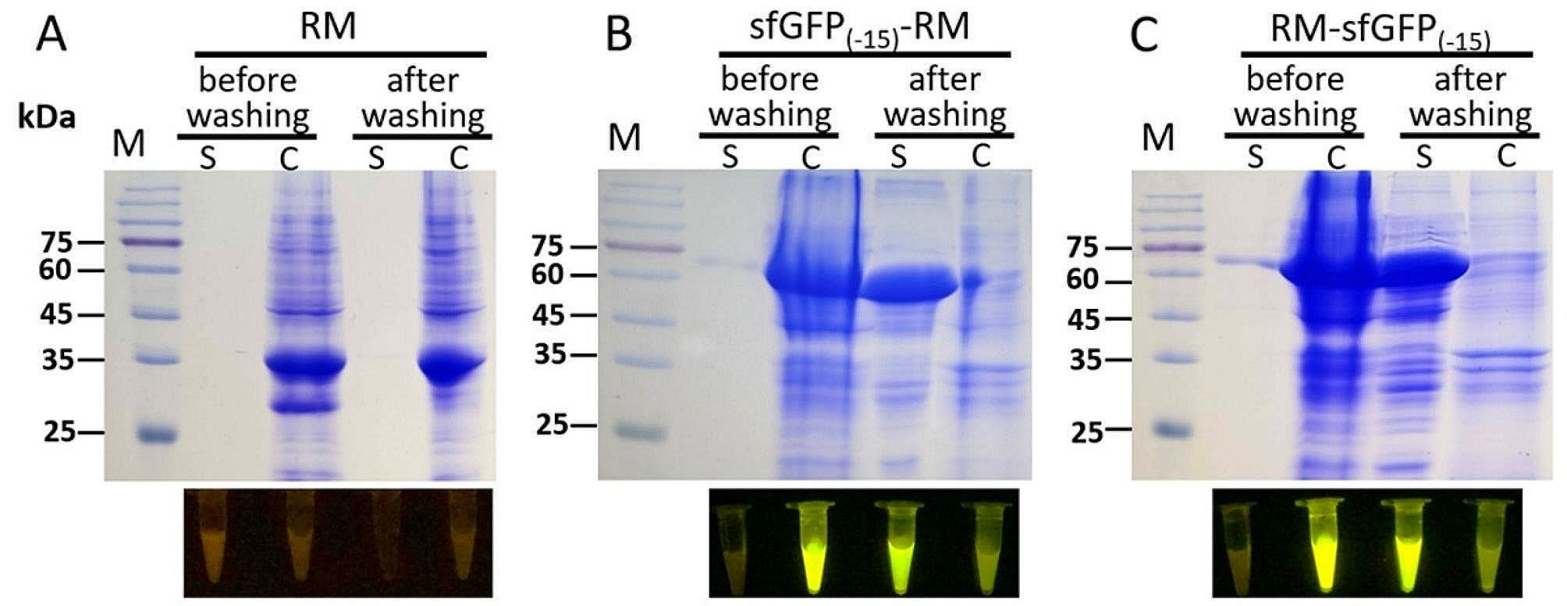
SDS-PAGE to analyze the sfGFP_(−15)_-mediated secretion expression of RM lipase. (A) the recombinant expression of RM lipase in *E. coli*; (B) the recombinant expression of RM lipase with sfGFP_(−15)_ fused to its N-terminus; (C) the recombinant expression of RM lipase with sfGFP_(−15)_ fused to its C-terminus. M. protein molecular weight marker (the size of each band was indicated on the left). S stands for the supernatant and C stands for the cells

To achieve secretion expression of RM lipase, its ORF (Genbank Accession No. AKA60076.1) including the propeptide coding sequence was cloned into pET23a-sfGFP_(−15)_ (Fig. 2A) and transformed to *E. coli* BL21 (DE3), followed by spreading on LB plates supplemented with ampicillin and incubating at 37 °C overnight. The colonies displayed obvious green fluorescence under blue light due to leaking expression of the fusion protein (Fig. 2B). The green colonies were inoculated into shake flasks for the expression of the target protein. The cell culture displayed bright green fluorescence. The result of SDS-PAGE indicated a single band consistent with the predicted molecular weight of the target protein was detected in the supernatant (Fig. 3B, C). On the contrary, no obvious band was detected in the supernatant of the cell culture with RM lipase expressed using intracellular expression vector pET23a-RM (Fig. 3A). Although only a small portion of the target protein was secreted, the target protein remained in the cells was translocated into the supernatant after incubated in PBS at 4 °C overnight (Fig. 3B, C). On the other hand, the intracellularly expressed RM lipase was remained in the cells after washing (Fig. 3A). Consistent with the result of SDS-PAGE, the fluorescence was transfer to the washing buffer after incubated with PBS (Fig. 3B, C). These results proved that sfGFP_(−15)_ mediated the secretion expression of RM lipase successfully. It worth noting that the secretion expression was achieved with sfGFP_(−15)_ fused to either N- or C-terminal of RM lipase, which implied that sfGFP_(−15)_ mediated secretion through a mechanism different from conventional signal peptides.

The previous reports indicated that RM lipase is synthesized as a precursor that includes a 70-amino acid propeptide before the 269 amino-acid residues of the mature enzyme. This propeptide is important for the correct folding of RM lipase [23–25]. In the present study, the specific activity of RM lipase was 8016.9 U/g while the specific activities of RM-sfGFP_(−15)_ and sfGFP_(−15)_-RM were 4153.7 U/g and 2453.3 U/g, respectively. As the molecular weight of RM lipase and the fusion protein were approximately 36.8 kDa and 64.5 kDa, the specific activity of RM lipase was 295.6 U/µmol and 258.6 U/µmol for RM-sfGFP_(−15)_, 157.3 U/µmol for sfGFP_(−15)_-RM. This result suggested that sfGFP_(−15)_ affected the activity of RM lipase slightly, but inhibited the propeptide to exert its function dramatically. Therefore, sfGFP_(−15)_ fused to the C-terminus of RM lipase (RM-sfGFP_(−15)_ ) was used for the further study.

### The Effect of sfGFP_(−15)_ Tag to the Activity of RM Lipase to Various Esters

The activity of RM lipase and RM- sfGFP_(−15)_ to commonly used chemical materials, such as CHCM, CHCI, DBP, DEHP, and DCHP were tested. The result indicated all of them can be catalyzed by both RM lipase and RM-sfGFP_(−15)_. RM lipase demonstrated 2.0 and 1.5-fold higher activity to plasticizers, DBP, DEHP, and DCHP than RM-sfGFP_(−15)_. On the contrary, RM-sfGFP_(−15)_ showed slightly higher activity to drug precursors, such as CHCM and CHCI (Supplementary Table 1). This result indicated the effects of sfGFP_(−15)_ to RM lipase vary with each chemical.

### Optimization of the Secretion Expression of RM Lipase

**Fig. 4:**
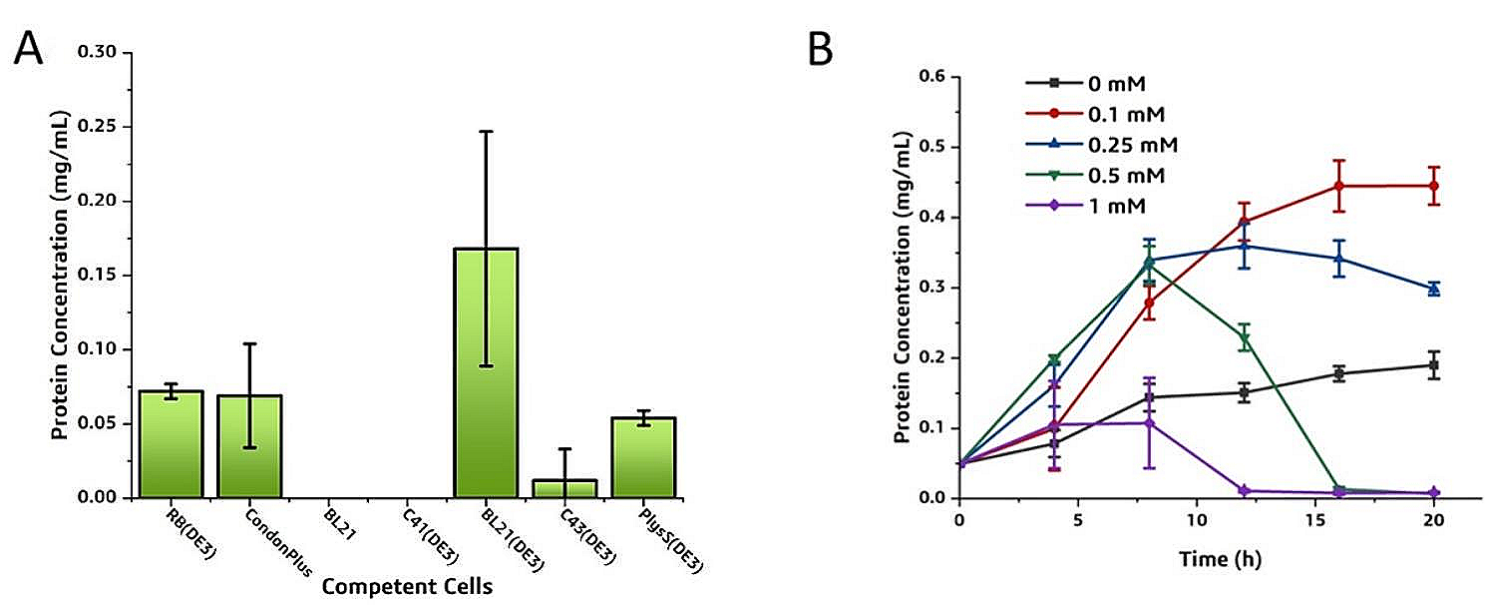
Optimization of the RM-sfGFP_(−15)_ expression condition. (A) the expression level of RM-sfGFP_(−15)_ with different hosts; (B) the expression level of RM-sfGFP_(−15)_ after induced with different concentrations of IPTG. Three parallel cell cultures were cultivated and standard deviations are indicated

Because sfGFP has absorbance at 488 nm, we plot a standard curve using the concentration of RM-sfGFP against the fluorescence intensity of the RM-sfGFP at 488 nm (Supplementary Fig. 1). Consequently, the concentration of the recombinant protein can be quantitively analyzed through measuring OD_488_ of the cell culture directly, which facilitated the expression condition optimization. The secretion expression of RM lipase with different *E. coli* expression strains were investigated and the result indicated the highest yield was obtained with *E. coli* BL21(DE3) (Fig. 4A). Therefore, it was chosen as the host for further studies. Next, the induction condition was optimized. The highest fluorescence intensity was obtained after 15 h of induction using IPTG of 0.1 mM (Fig. 4B), with a yield of the target protein reached 0.168 g/L.

### High-density Fermentation to Generate RM-sfGFP_(−15)_

**Fig. 5:**
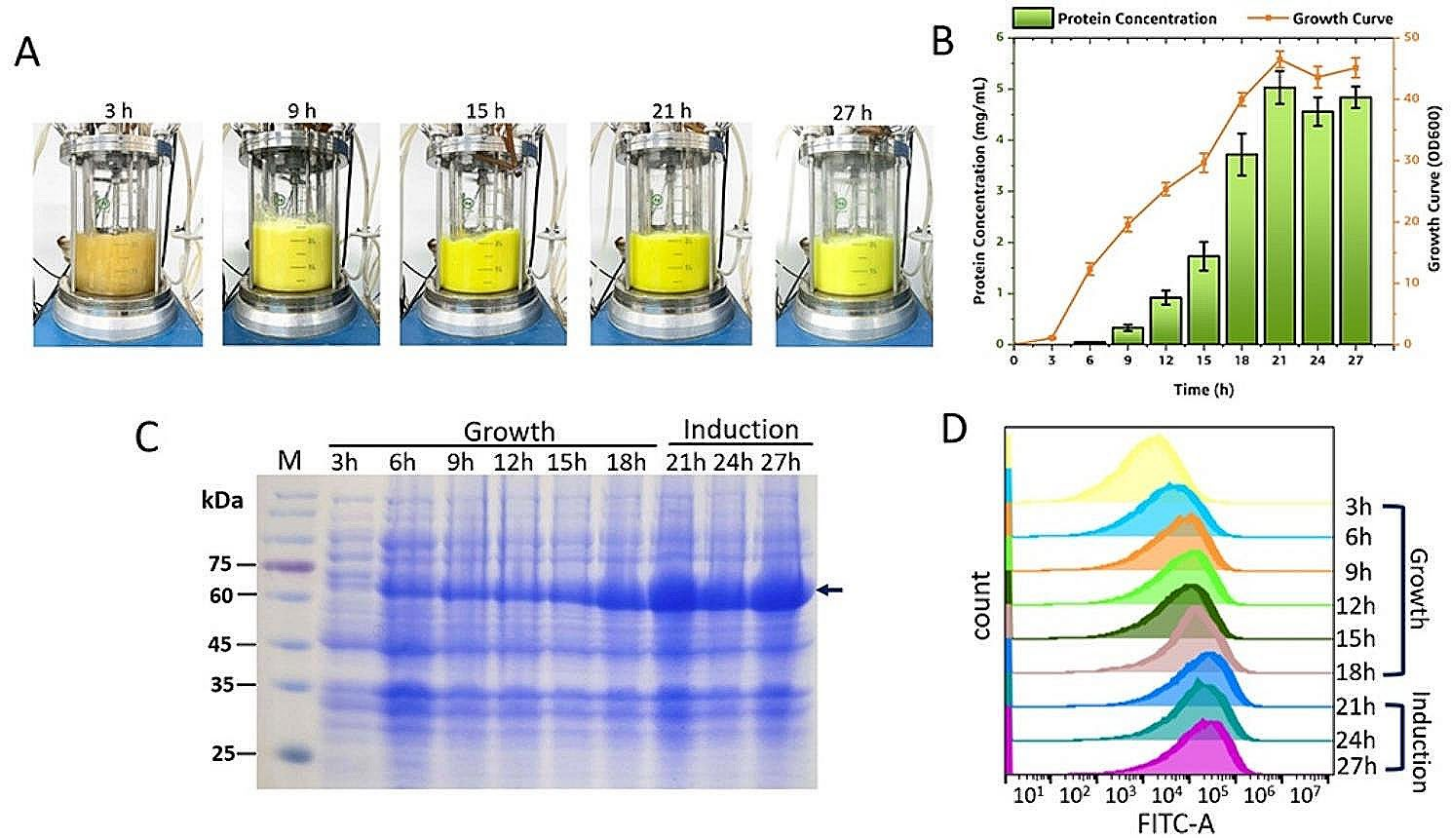
The 5-L high-density fermentation of RM-sfGFP_(−15)_. (A) the color change of the cell culture during the fermentation; (B) cell density and the fluorescent intensity of the target protein during the fermentation; (C) SDS-PAGE to analyze the expression of the target protein during the fermentation (the target protein is indicated with a black arrow). Three samples were collected at each time point and standard deviations are indicated. M. protein molecular weight marker (the size of each band was indicated on the left); (D) FACS to analyze the fluorescent intensity of the cells during the fermentation

The RM lipase was also prepared with high-density fermentation. The color change of the cells during the whole process of the fermentation was visible (Fig. 5A). The green fluorescence intensity of the cell culture increased gradually during the first 18-h cell growth phase and reached maximum after 3 h of induction (Fig. 5B). The results of SDS-PAGE and FACS confirmed this result (Fig. 5C and D, Supplementary Fig. 2).

**Fig. 6:**
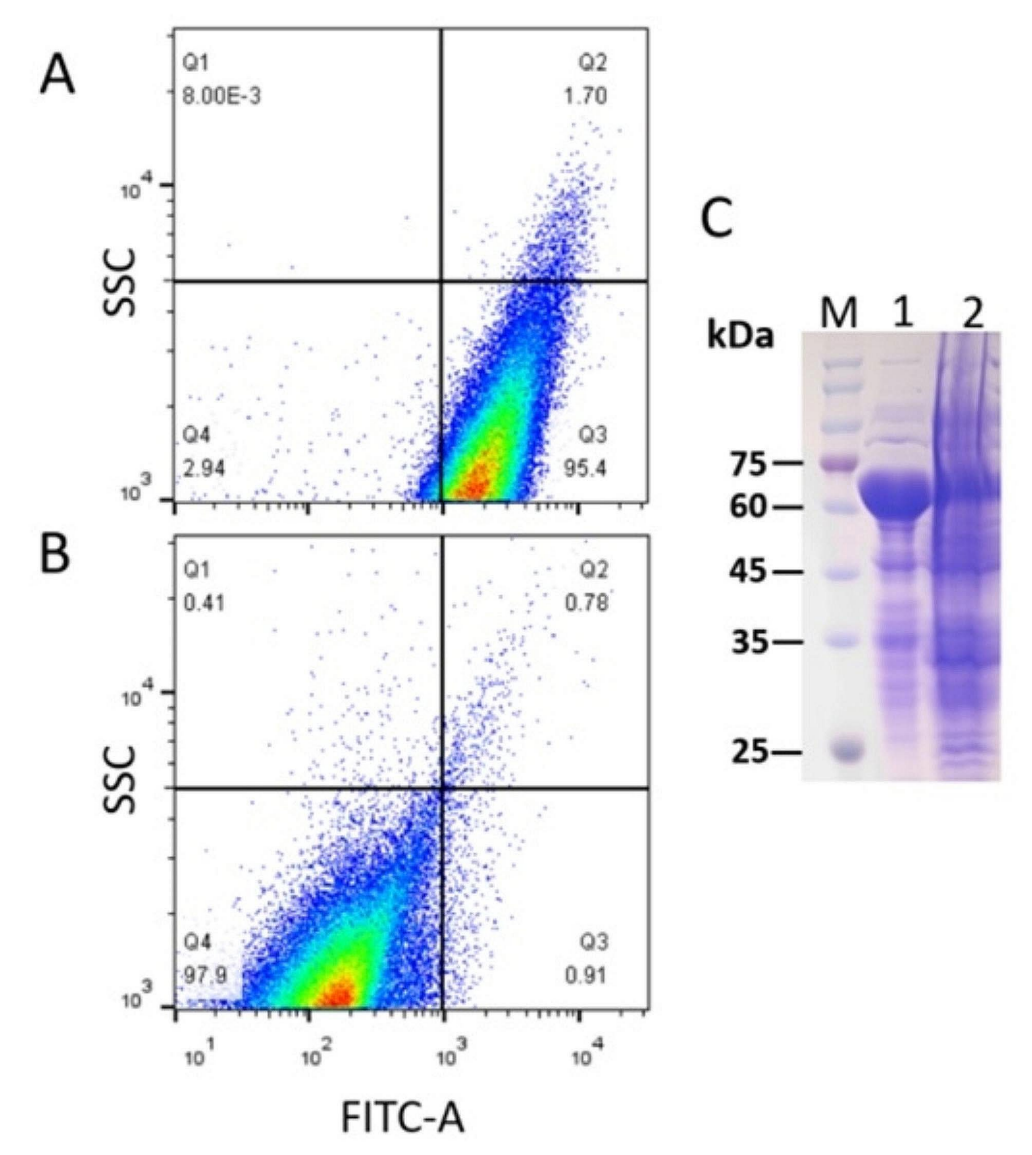
FACS and SDS-PAGE to analyze the of fluorescent intensity of the cells. (A) cells collected before washing with PBS overnight; (B) cells collected after washing with PBS overnight. (C) SDS-PAGE of the cells from A (lane 1) and B (lane 2). M. protein molecular weight marker (the size of each band was indicated on the left)

After the fermentation, the cells were collected and RM-sfGFP_(−15)_ was washed off from the cells. The yield of the target protein was approximately 5.1 mg/mL. The result of FACS indicated the shape of the cells remained contact after washing with PBS while the fluorescence intensity decreased dramatically (Fig. 6A and B), which suggested that the target protein was released to the buffer without causing cell disruption. The result of SDS-PAGE also indicated that most of the target protein was translocated into PBS after washing (Fig. 6C). Therefore, the target protein was separated from the endogenous proteins through centrifugation and the supernatant was utilized for immobilization directly.

### The Immobilization of RM-sfGFP_(−15)_

**Fig. 7:**
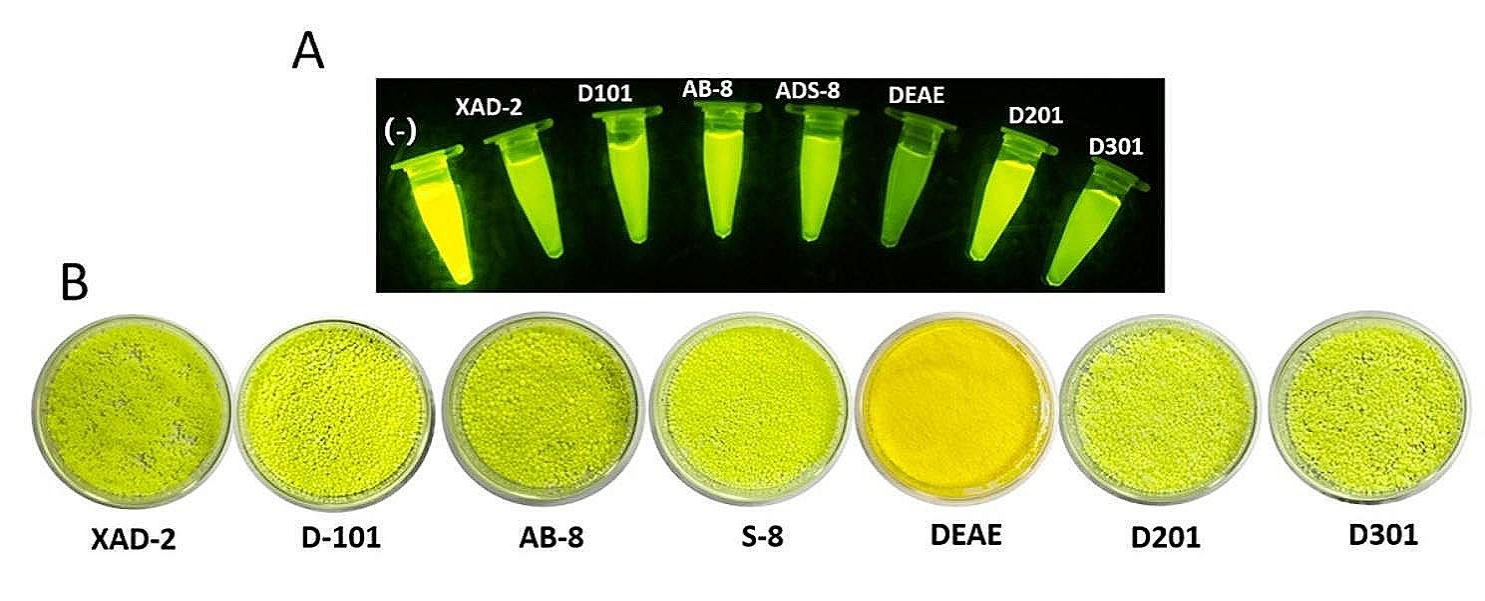
The immobilization of RM-sfGFP_(−15)_ with various resins. (A) the enzyme left in the supernatant after the immobilization. The types of the resins are label on the top of the tubes; (B) immobilized RM-sfGFP_(−15)_ fixed on various support materials

RM-sfGFP_(−15)_ was immobilized with three types of resins, including nonpolar macroporous, polar macroporous, and anion-exchange resins (Table 1). All of the resins were able to fix RM-sfGFP_(−15)_.The fluorescence intensity of the enzyme solution decreased obviously after immobilization and the resins were stained to bright green (Fig. 7A). Since the surface of RM-sfGFP_(−15)_ molecules were covered with negative charges, it is reasonable that anion-exchange resins, such as DEAE and D301 showed the highest immobilization capacity. Interestingly, DEAE demonstrated above 5-fold higher binding capacity to RM-sfGFP_(−15)_ in comparison with D301 resin. Approximately 364.5 mg of the target protein was immobilized by 1 g of DEAE resin. On the contrary, D201 resin demonstrated the lowest immobilization efficiency, with only 46.8 mg of the target protein was fixed (Table 1). The enzyme activity of RM-sfGFP_(−15)_ was inhibited to different degree after immobilization (Table 1). The enzyme activity of RM-sfGFP_(−15)_ decreased dramatically after fixed with DEAE resin, which was only approximately 2248.1 U/g (Table 1). On the other hand, RM-sfGFP_(−15)_ remained approximately 80% activity after fixed with D101 resin, which was 3626.2 U/g.

It is reported that KCl improves the activity of immobilized RM lipase [26]. The result of the present study indicated KCl improved 20.6% of the activity of RM-sfGFP_(−15)_ immobilized with D101. On the contrary, KCl affected the binding of RM-sfGFP_(−15)_ to DEAE resin significantly and most of the target protein failed binding to DEAE resin in the presence of KCl. Therefore, D101 resin was chosen as the supporting material for the immobilization of RM-sfGFP_(−15)_. The immobilized enzyme was named as RM-sfGFP_(−15)_ IM.

**Table 1:** the resins used in the present study and their immobilization efficiency

Resin	Type of the resin	The material of the resin	The amount (mg) of protein fixed per g of the resin	Binding efficiency	The specific activity (U/g of the protein)
XAD-2	Nonpolar macroporous	Poly (styrene divinylbenzene)	61 ± 4.1	48.8 ± 2.9	1921.8 ± 128.3
D101	Nonpolar macroporous	polystyrene	69.5 ± 5.4	55.6 ± 6.8	3626.2 ± 96.5
AB-8	Weakly polar macroporous	polystyrene	70.2 ± 2.3	56.1 ± 2.4	3452.3 ± 132.5
S-8	weakly polar macroporous	polystyrene	55.9 ± 4.8	44.7 ± 1.6	2538.2 ± 78.6
DEAE	Weak-basic anion-exchange	cellulose	364.5 ± 36.8	87.9 ± 2.5	2248.1 ± 113.5
D201	strong-basic anion-exchange	polystyrene	46.8 ± 3.5	37.5 ± 1.8	3046.2 ± 125.1
D301	Weak-basic anion-exchange	polystyrene	70.5 ± 4.1	56.4 ± 4.4	3125.3 ± 175.3

The results are presented as the means ± SDs of three independent experiments.

### The Features of RM-sfGFP_(−15)_ IM

**Fig. 8:**
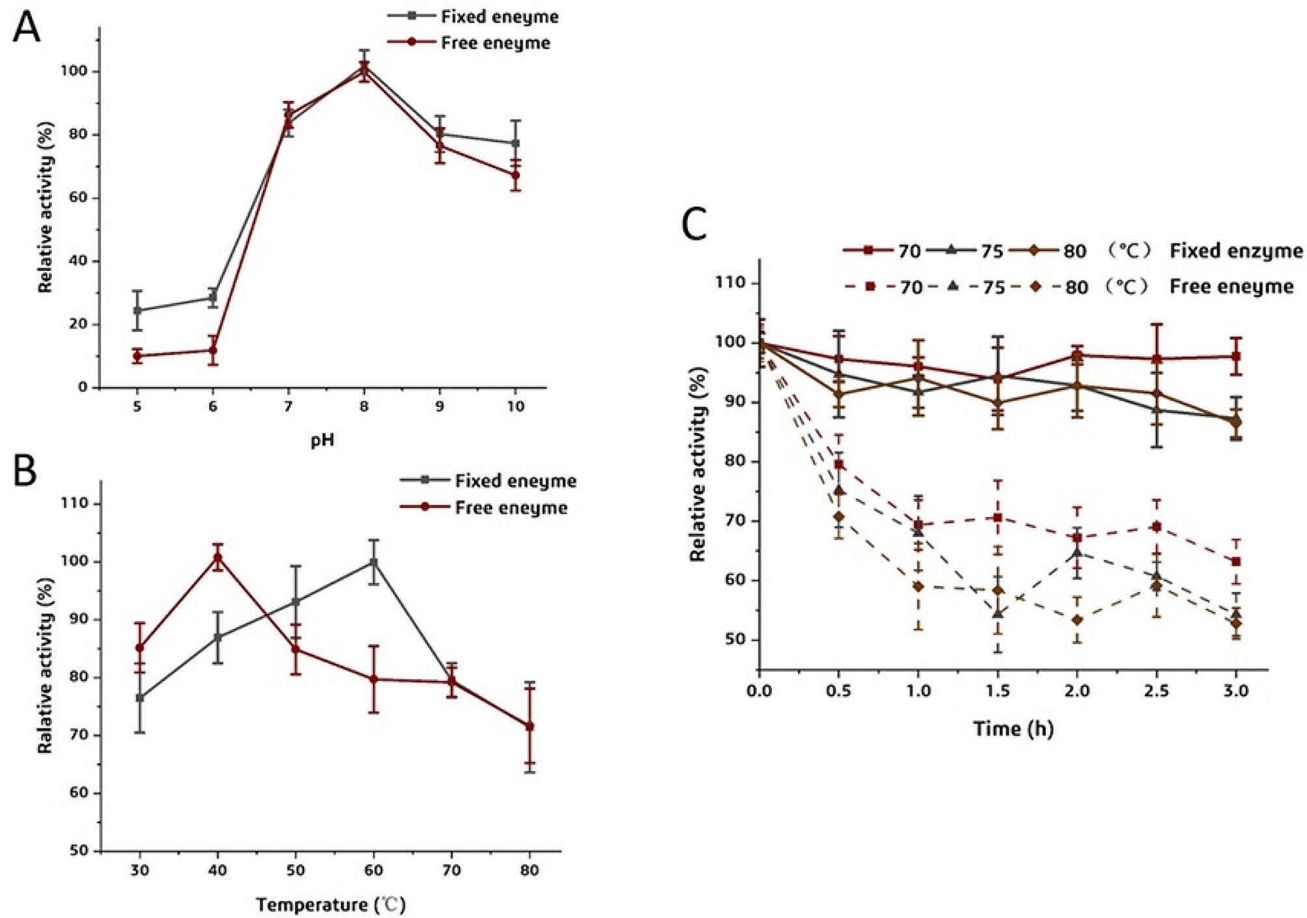
The characteristics of RM-sfGFP_(−15)_ IM in comparison with the free RM-sfGFP_(−15)_. (A) the optimum pH of free and immobilized RM-sfGFP_(−15)_; (B) the optimum temperature of free and immobilized RM-sfGFP_(−15)_; (C) The thermostability of free and immobilized RM-sfGFP_(−15)_. Each reaction was performed in triplicate and standard deviations are indicated

The optimal pH of RM-sfGFP_(−15)_ IM was pH 7.5, which was consistent with the free enzyme (Fig. 8A). However, the optimal temperature of RM-sfGFP_(−15)_ IM was 60 ℃, which was much higher than the free enzyme (Fig. 8B). In addition, the thermostability of RM-sfGFP_(−15)_ improved significantly after immobilization. The fixed enzyme retained above 90% of its activity after incubation at 80 ℃ for 3 h. On the contrary, the free enzyme lost half activity under this condition (Fig. 8C).

**Fig. 9:**
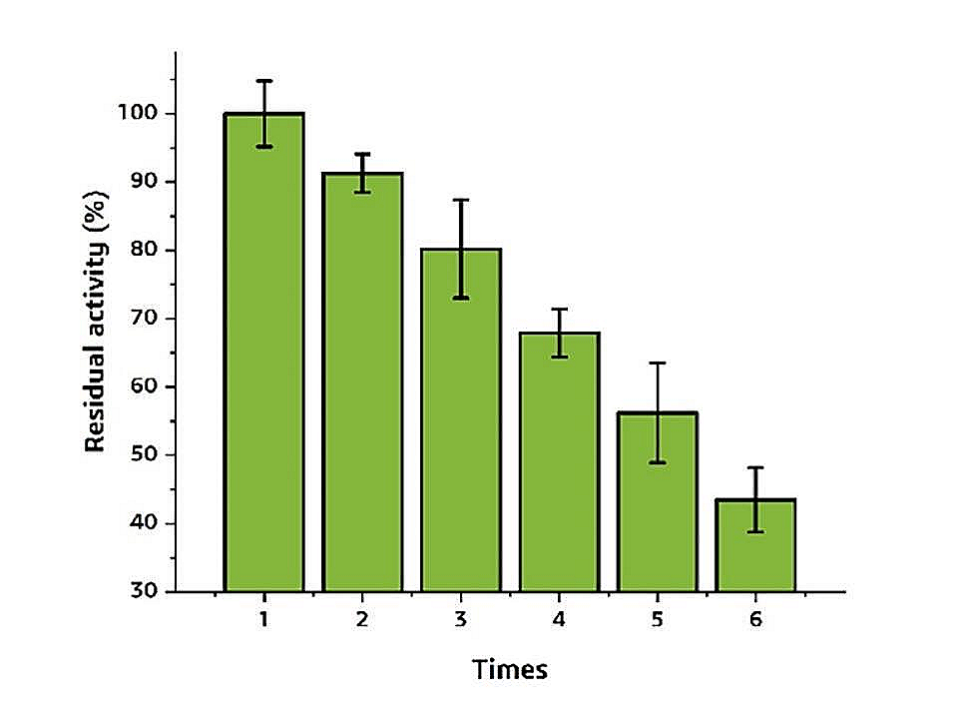
The reusability of RM-sfGFP_(−15)_ IM. The initial activity of RM-sfGFP_(−15)_ IM was taken as 100%. The experiments were performed in triplicates and standard deviations are indicated

The immobilized enzyme can be reused for the catalytic reaction and save the cost in industry. Therefore, the reusability of RM-sfGFP_(−15)_ IM was investigated. The conversion efficiency of RM-sfGFP_(−15)_ IM decreased slightly and remained approximately 91.3% activity at the second round of reaction. And then the conversion efficiency decreased gradually with the repetitive use. After 6 repeats, only 43.5% of the initial activity was preserved (Fig. 9). During the whole progress, the RM-sfGFP_(−15)_ IM remained green color. The stability of the immobilized enzyme was tested after stored at 4 ℃ for 5 months. The result indicated that the immobilized enzyme remained approximately 71.2% of its activity.

### Conversion of TAG to DAG with RM-sfGFP_(−15)_ IM

**Fig. 10:**
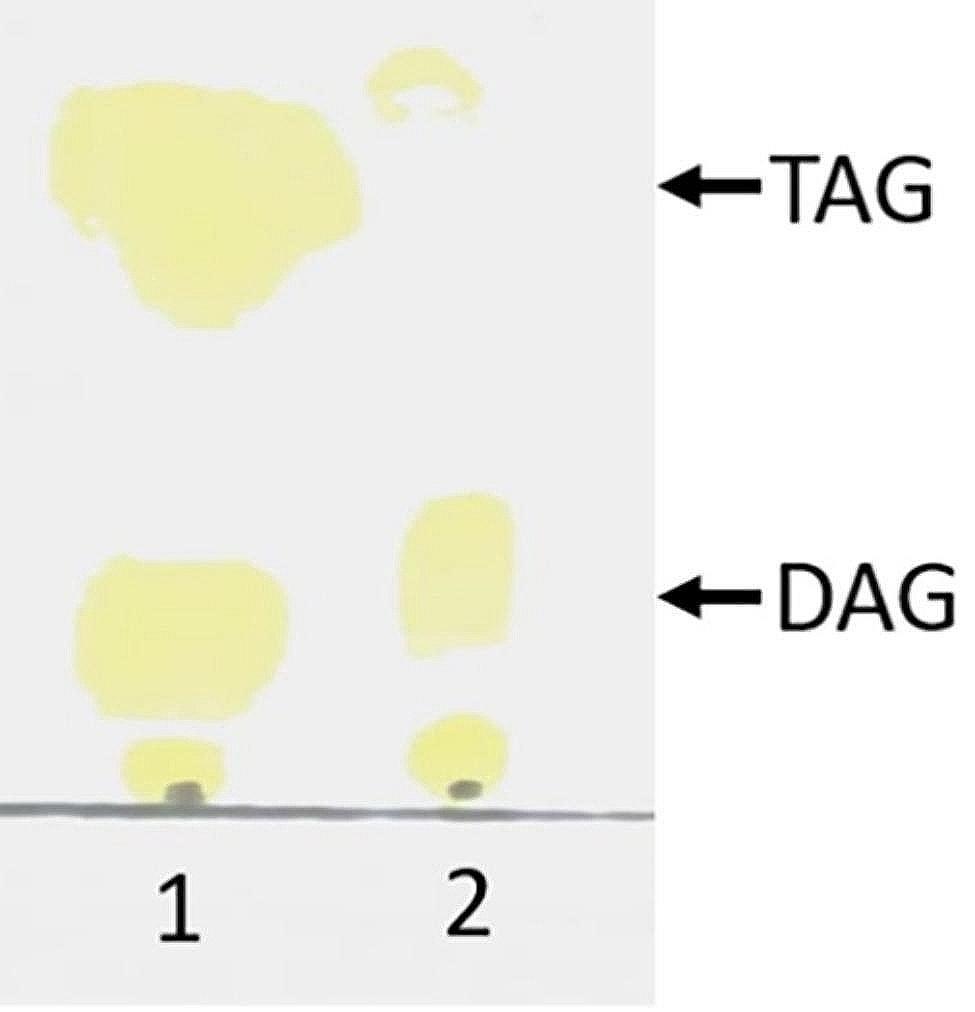
TLC to analyze the DAG generated by glycerolysis of soybean oil with RM-sfGFP_(−15)_ IM.1: RM-sfGFP_(−15)_ IM; 2: DAG Standard

Previous reports applied RM lipase to synthesize DAG by glycerolysis of soybean oil in a solvent-free system [27, 28]. The present study indicated that RM-sfGFP_(−15)_ IM was able to catalyze this reaction. DAG was generated at 3-h reaction at 50 °C (Fig. 10).

## Discussion

It reported that sfGFP and its mutants promoted the secretion expression of the foreign proteins [17]. The fusion protein was translocated from cytoplasm to the inner membrane, subsequently transporting to the outer membrane, and eventually the extracellular medium. Later, Zhang et al., applied this sfGFP mediated secretion system to gram-positive microbes and achieved secretary expression of phospholipase D in *Bacillus subtilis* successfully [29]. However, the mechanism remains unclarified. The previous study indicated that enhanced green fluorescence protein (eGFP) fused to the 218 C-terminal amino acids of α-hemolysin (HlyAc) was unable to be secreted by the type I secretion system and stalled the secretion of HlyAc, presumably because folded protein blocked the translocation [16]. The present study indicated sfGFP_(−15)_ promotes the secretion of the target proteins fused to either N- or C-terminus. Therefore, we deduced that sfGFP and its mutants mediated the secretion through a noncanonical mechanism. Moreover, majority of the fusion protein was still retained in the cells and was separated from the cells through washing with PBS, which is also different from the conventional secretion expression. The previous study demonstrated the net negative charges of sfGFP mutants play important role in promoting the secretion expression of the fusion proteins. Consistent with this theory, RM lipase with a relatively low pI (approximately 4.82) demonstrated higher secretion efficiency than *Thermomyces lanuginosus* (TL) lipase (pI 5.23 ), which can also be expressed with this system efficiently, but cannot be washed off from the cells efficiently (data not shown). Another reason why RM lipase can be secreted efficiently may relate with the catalytic activity of RM lipase. This lipase may disturb the phospholipid bilayer of the cells and improve the translocation of the proteins from inner membrane to periplasm. Therefore, it is perspective to achieve successful expression of more lipases, phospholipases, esterase etc. with this system.

In most cases, RM lipase requires immobilization before used as industrial biocatalyst [3]. The binding of lipases on suitable supports allows the re-use of the biocatalysts and save the cost of the enzymes. Moreover, immobilization improves the enzyme activity, selectivity and decreases inhibition occasionally. RM lipase is an interfacial enzyme performs its function at the interface of drops of insoluble substrates (oils or fats). These lipases display low activity on water soluble, monomeric substrates in homogenous aqueous solution. As the substrate concentration increases and exceeds its critical micelle concentration, the activity increases dramatically. This phenomenon is called interfacial activation [30–32]. According to the structures of lipases, the interfacial activation mechanism was clarified. There is a short polypeptide chain called lid (or flap) exists in a lipase. In the closed form, it isolates the active center from the surrounding hydrophilic environment. When the lid moves to an open form, a large hydrophobic pocket and the active center is exposed to the environment, which becomes strongly adsorbed on any hydrophobic surface [33–36]. According to this theory, the well-defined hydrophobic support surfaces may resemble the hydrophobic drops of insoluble natural lipase substrates. Therefore, hydrophobic supports were considered as good support for RM lipase [37]. The lipase is adsorbed on strongly hydrophobic solid surfaces and exhibits a dramatic enhanced esterase activity in the absence of additional hydrophobic interfaces. Moreover, these supports promote highly selective adsorption of lipases, at very low ionic strength, from impure protein extracts. In this study, we chose 6 resins with different properties as the supporting material. The result indicated the fusion protein was fixed with DEAE resin at a high capacity and the binding was very stable. It is reasonable since RM lipase-sfGFP_(−15)_ carries high net negative charges. However, the enzyme activity was significantly jeopardized after the binding. The clue lays in fact that the DEAE is a cellular-based hydrophilic material, which fails to provide a hydrophobic surface. The support of the RM IM from Novozyme is Duolite ES 562, a weak anion-exchange resin based on phenol–formaldehyde copolymers [9]. In the present study, the weak anion-exchange polystyrene resin D301 also showed a high loading capacity and activity. However, D101 and D201 resins also derived from polystyrene demonstrated an even better binding capacity. Although the activity of RM lipase was inhibited slightly after fixed, the thermostability of the fixed enzyme improved obviously. Moreover, these resins are cheap (approximately 10 dollar/kg) and the whole procedure is straightforward. Therefore, preparing immobilized RM lipase with polystyrene-based resins is feasible. It is reported that octyl–glyoxyl agarose is an ideal support for lipase because the enzyme can be immobilized on this support stability through interfacial interaction [38–40]. It is a good candidate and worths further study.

The commercial liquid RM and immobilized RM was prepared with *A. oryzae*, a Generally Recognized as Safe (GRAS) strain [41]. However, the yield of RM lipase still needs to be improved. In 2022, Li et al. increased the yield of recombinant RM lipase to 550.0 U/mL through morphology control and optimization of the fermentation process [42] Another problem with this system is the extracellular proteins of *A. oryzae* are abundant. Therefore, the enzyme product contains many *A. oryzae* endogenous proteins, which affects the industrial application of the enzyme. Lipase can also be prepared with *Pichia pastoris*, which is also GRAS [43, 44]. Moreover, the strong secretary expression capacity of *P. pastoris* lead to a high purity of the product. However, the yield of RM lipase with this system is also less than 1 g/L [12, 45]. Furthermore, the fermentation period of *P. pastoris* is more than a week, which is much longer than *E. coli* and hence, increase the cost of the recombinant RM lipase. The method in the present study overcame the low secretion efficiency of *E. coli* recombinant expression system [46]. The yield of the target protein reached 5.1 g/L and the pure enzyme can be washed off from cells without complex extraction process. Considering the high activity of RM, the low-cost RM-sfGFP_(−15)_ prepared in the present study is an excellent source for biodiesel conversion. It can also be applied in pharmaceutical industry because its high activity and the protein can be removed easily during the downstream product-purification process [47, 48]. In addition, the membrane filtration in oil processing industry can also remove RM-sfGFP_(−15)_ completely. However, we don’t recommend to add RM-sfGFP_(−15)_ in food directly because *E. coli* generates endotoxin [49]. Although most cells retained contact during the PBS-washing step. Cells disrupted during fermentation and downstream preparation can still release endotoxin and contaminate the enzyme. Therefore, the enzyme needs to be purified before applied in food industry, which increased the cost of the product dramatically.

In summary, large-scale preparation of RM lipase with a visually accessible, cost-effective way was achieved in this study. Moreover, this sfGFP-mediated secretion expression system also demonstrated great potential in industrial preparation of other lipases.

### Electronic Supplementary Material

Below is the link to the electronic supplementary material.

Supplementary Material 1

Supplementary Material 2

## Data Availability

No datasets were generated or analysed during the current study.
