# Supplementary material for: A Whole-Process Visible Strategy for the Preparation of Rhizomucor miehei Lipase with Escherichia coli Secretion Expression System and the Immobilization
Source: Microb Cell Fact. 2024 May 27;23:155. doi: 10.1186/s12934-024-02432-y (PMC11129466; doi:10.1186/s12934-024-02432-y)
Supplement: Supplementary file 1 — Supplementary Material 1 [file 12934_2024_2432_MOESM1_ESM.docx]

**Supplementary Figure**


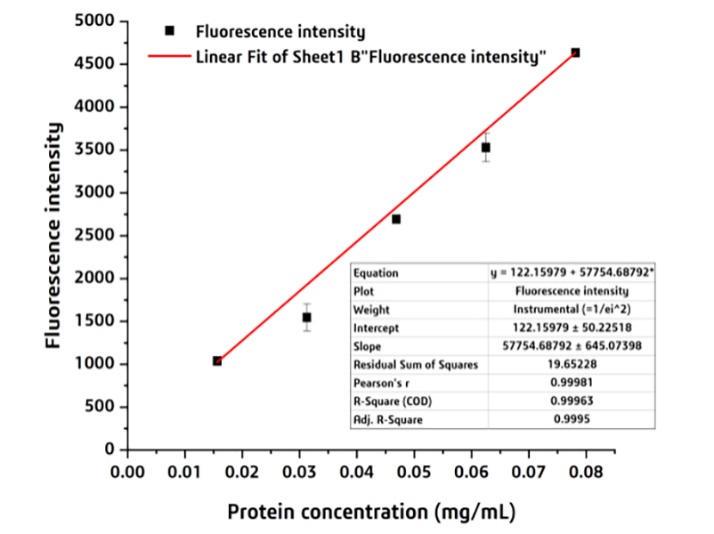


Supplementary Fig. 1 the standard curve of calculation the protein concentration through measuring the fluorescence intensity at 488 nm.


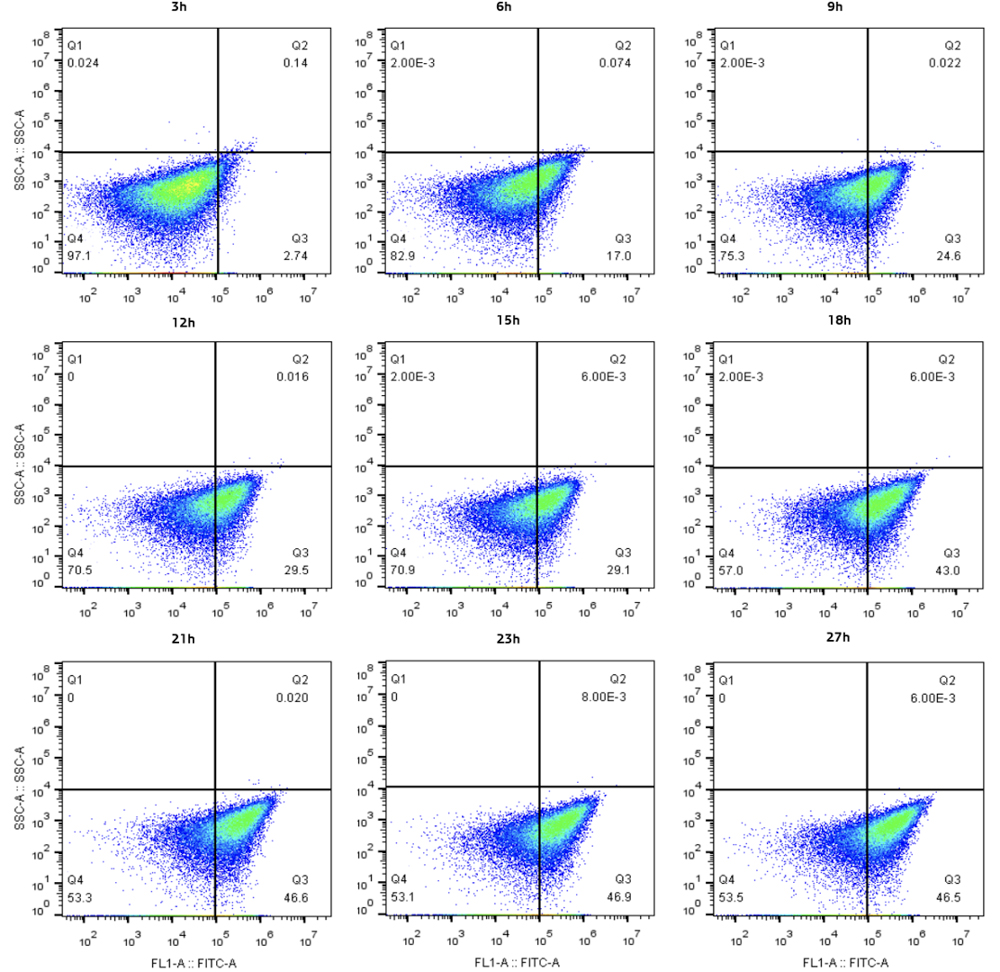


Supplementary Fig. 2 FACS to analyze the fluorescent intensity of the cells during the fermentation.

**Supplementary Table**

**Supplementary Table 1 The activity of RM lipase and** **RM-sfGFP_(-15)_ to different esters.**

| Substrate | Activity of RM-sfGFP_(-15)_ against to RM（%） |
| --- | --- |
| DBP | 71.3±6.3 |
| DEHP | 50.1±3.7 |
| DCHP | 69.4±7.8 |
| CHCM | 102.4±2.9 |
| CHCI | 105.8±1.8 |

The results are presented as the means±SDs of three independent experiments.
