# Supplementary material for: A Whole-Process Visible Strategy for the Preparation of Rhizomucor miehei Lipase with Escherichia coli Secretion Expression System and the Immobilization
Source: Microb Cell Fact. 2024 May 27;23:155. doi: 10.1186/s12934-024-02432-y (PMC11129466; doi:10.1186/s12934-024-02432-y)
Supplement: Supplementary file 2 — Supplementary Material 2 [file 12934_2024_2432_MOESM2_ESM.docx]

**Full uncropped Gels images**

**
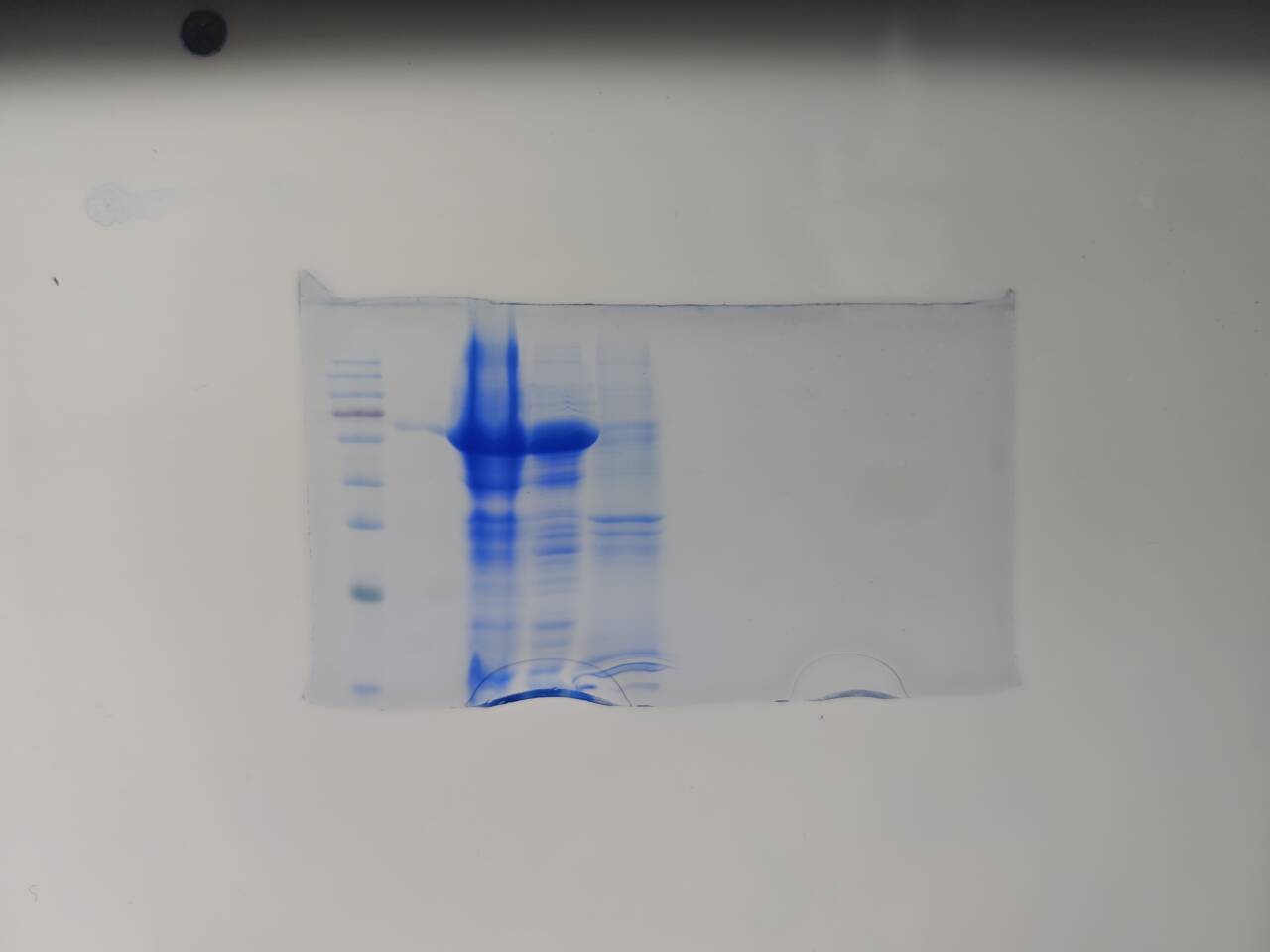

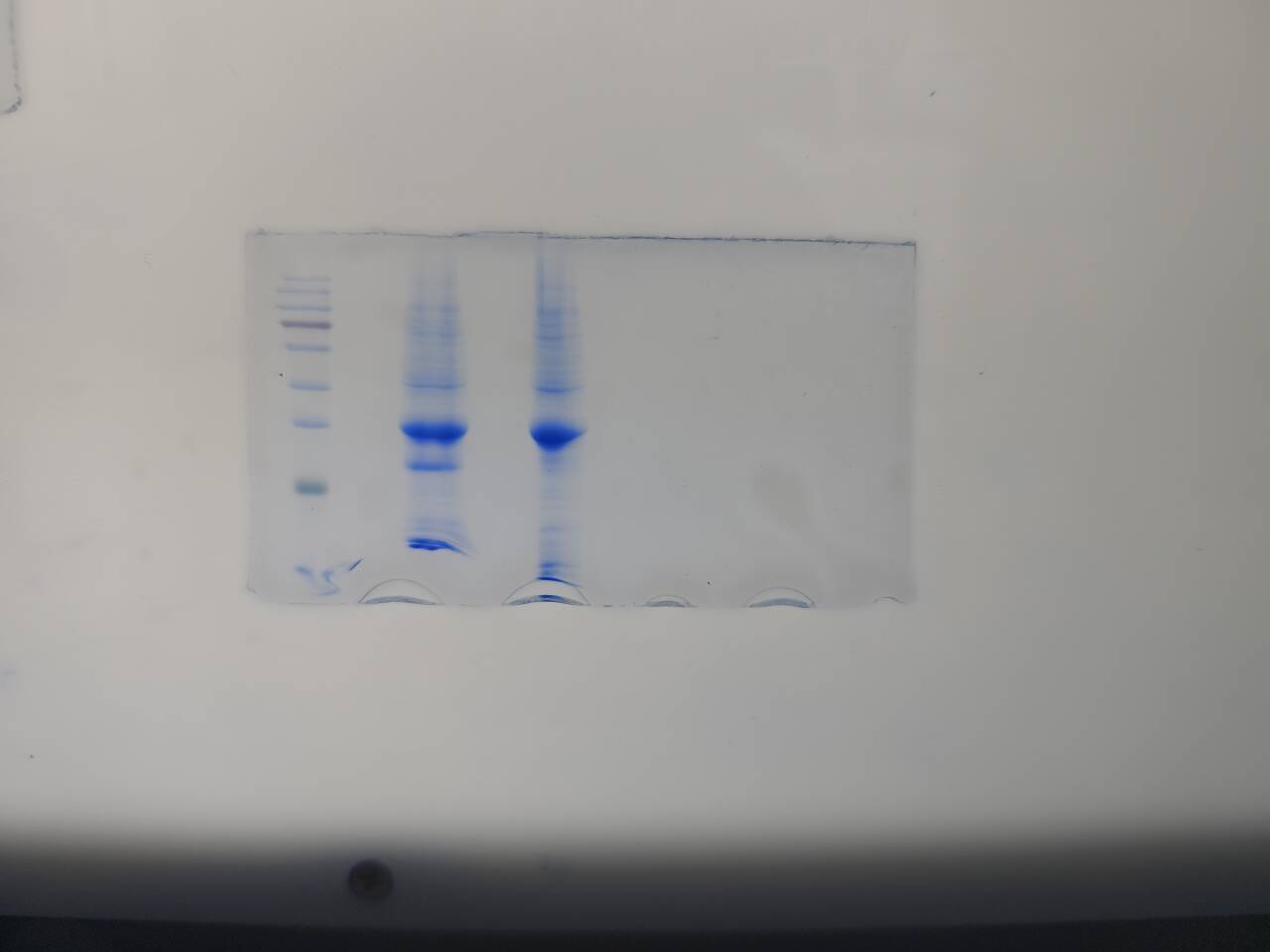

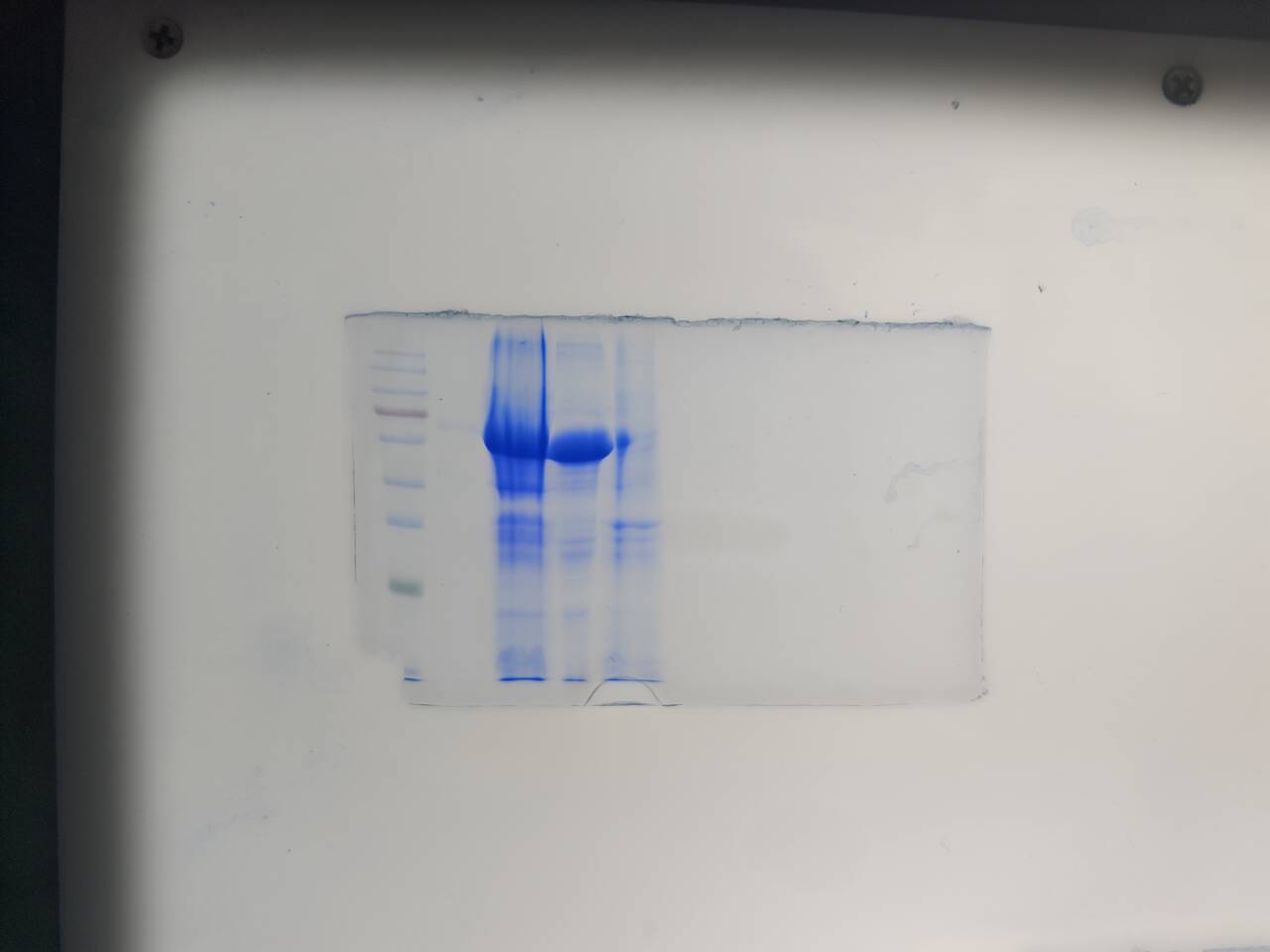
**

**Full uncropped Gels images-Figure 3(manuscript)**

**
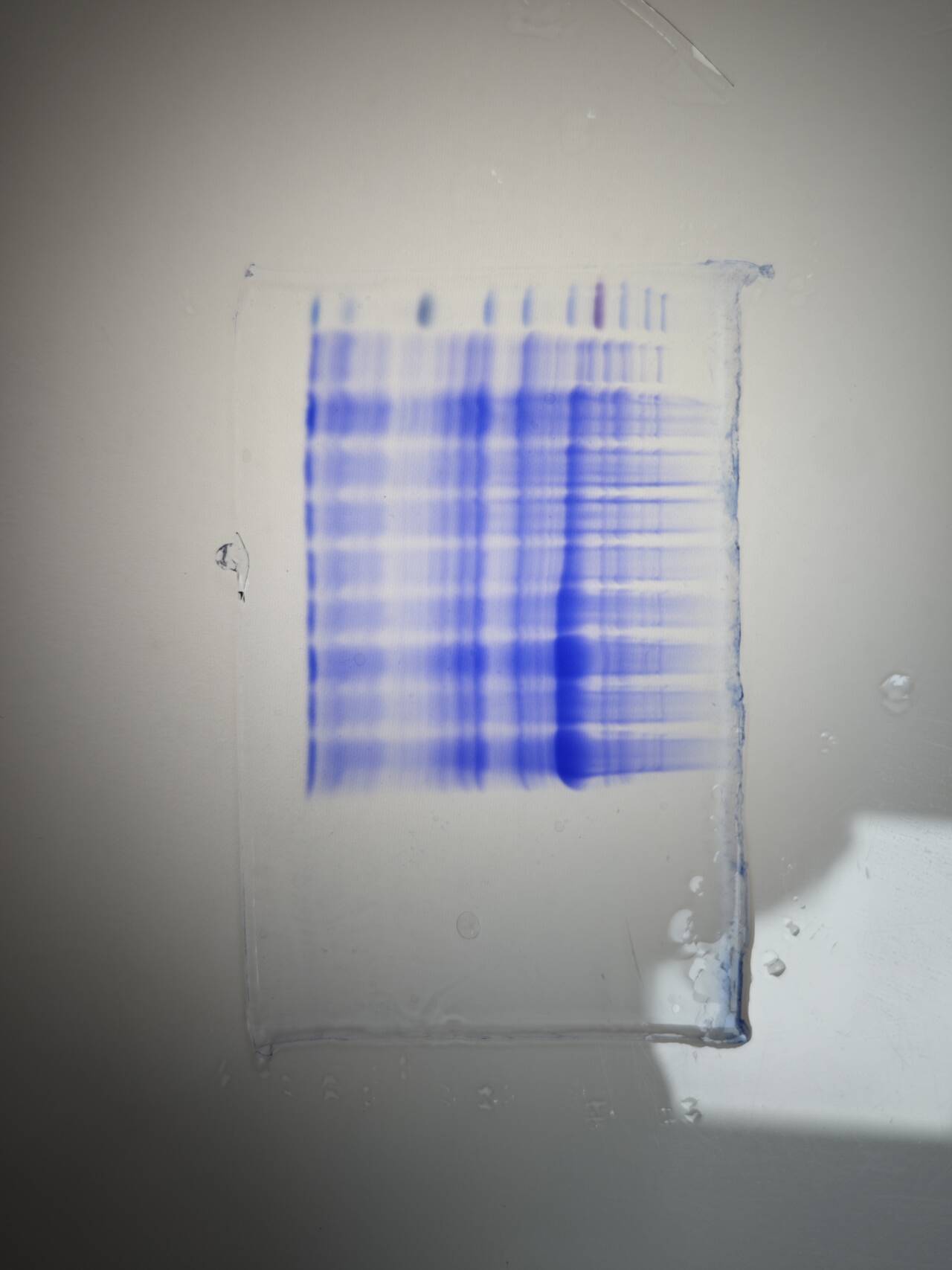
**

**Full uncropped Gel image-Figure 5(manuscript)**

**
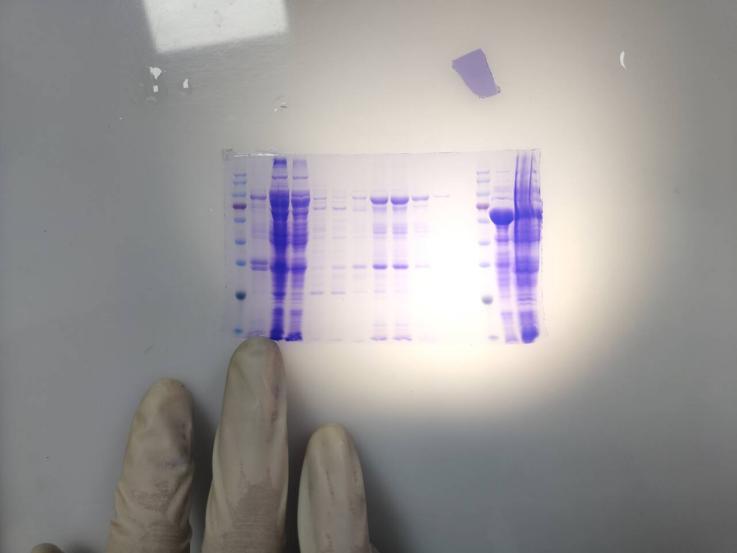
**

**Full uncropped Gel image-Figure 6(manuscript)**
